# Interpreting the Process behind Endemism in China by Integrating the Phylogeography and Ecological Niche Models of the *Stachyridopsis ruficeps*

**DOI:** 10.1371/journal.pone.0046761

**Published:** 2012-10-02

**Authors:** Huatao Liu, Wenjuan Wang, Gang Song, Yanhua Qu, Shou-Hsien Li, Jon Fjeldså, Fumin Lei

**Affiliations:** 1 Key Laboratory of Zoological Systematics and Evolution, Institute of Zoology, Chinese Academy of Sciences, Beijing, China; 2 Graduate School of the Chinese Academy of Sciences, Beijing, China; 3 Department of Life Sciences, National Taiwan Normal University, Taipei, Taiwan; 4 Center of Macroecology, Evolution and Climate, Natural History Museum of Denmark, Copenhagen, Denmark; University of Sydney, Australia

## Abstract

An area of endemism (AOE) is a complex expression of the ecological and evolutionary history of a species. Here we aim to address the principal drivers of avian diversification in shaping patterns of endemism in China by integrating genetic, ecological, and distributional data on the Red-headed Tree Babbler (*Stachyridopsis ruficeps*), which is distributed across the eastern Himalayas and south China. We sequenced two mtDNA markers from 182 individuals representing all three of the primary AOEs in China. Phylogenetic inferences were used to reconstruct intraspecific phylogenetic relationships. Divergence time and population demography were estimated to gain insight into the evolutionary history of the species. We used Ecological niche modeling to predict species’ distributions during the Last Glacial Maximum (LGM) and in the present. Finally, we also used two quantitative tests, an identity test and background test to assess the similarity of ecological niche preferences between adjacent lineages. We found five primary reciprocally monophyletic clades, typically separated approximately 0.2–2.27 MYA, of which three were deeply isolated endemic lineages located in the three AOEs. All phylogroups were detected to have undergone population expansion during the past 0.3 MY. Niche models showed discontinuous habitats, and there were three barriers of less suitable habitat during the LGM and in modern times. Ecoclimatic niches may diverge significantly even over recent timescales, as each phylogroup had a unique distribution, and unique niche characteristics. Vicariant events associated with geographical and ecological barriers, glacial refuges and ecological differentiation may be the main drivers forming the pattern of endemism in China.

## Introduction

The area of distribution of a species is a complex expression of its ecological and evolutionary history [1]–[2]. Integrating phylogeographic and ecological data have provided new insights on speciation and species’ distribution dynamics [3]–[7]. Phylogeographic studies suggest that geological, climatic, ecological process and other factors all play roles in molding population structure, eventually leading in some cases to reproductive isolation and speciation [7]. Over the past two decades, the use of genetic markers to identify evolutionarily distinct populations has become routine [8]; this technique has played an important role in describing the process of speciation [9]–[11], and revealed the presence of cryptic endemic species [12]. In evaluating the historical process of speciation, ecological data can complement phylogeographic research by providing multifaceted information about the origins, evolutionary history and present distribution of species or phylogroups [13]. Ecological data also shows enormous promise for elucidating how isolation, selection, and speciation directly or indirectly link to earth history [14]. One way to use these ecological data is in ecological niche modelling (ENM), which use collection sites and ecological data modeled in a Geographic Information System (GIS) framework to identify factors that have contributed to the divergence of terminal taxa [14]–[15]. Although controversy surrounds the extent to which niche dimensions have been conserved in a given group [16], ENM has the capacity to improve our understanding of patterns of endemism and can accelerate the discovery process for new species [4], [6]. During the last decade, research integrating these fields has become a powerful tool to address issues in evolution, biogeography, ecology and conservation biology [17]–[19].

**Figure 1:**
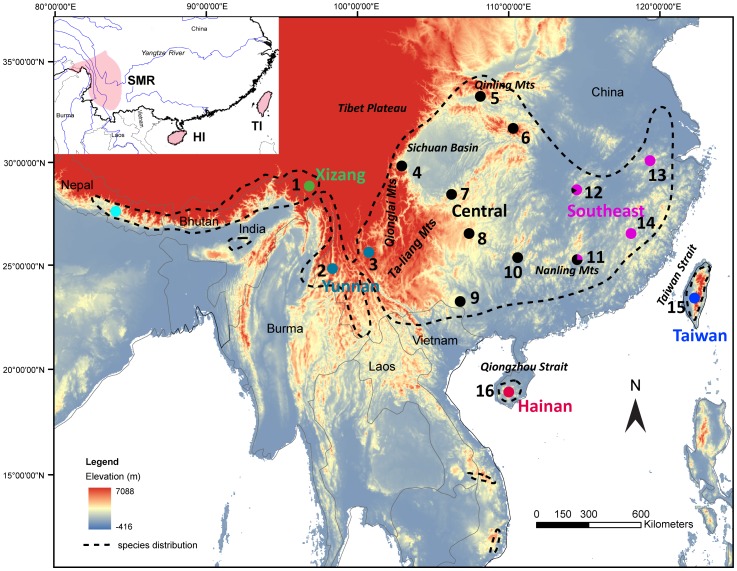
Sampling sites and the geographic distribution of *Stachyridopsis ruficeps* lineages. Sampling localities are indicated by dots, and the site numbers correspond to those in the Appendix. Each colour represents a lineage as identified in the phylogenetic trees. “–-” corresponds to the species’ distributions: *S. r. ruficeps* is located in E Nepal to NE India and SE Xizang; *S. r. bhamoensis* in W & NW Yunnan and NE Burma; *S. r. davidi* in C, E and S China, NW Laos and N Vietnam; *S. r. praecognita* in Taiwan; *S. r. goodsoni* in Hainan Island and *S. r. pagana* in S Vietman and S Vietnam [39]–[40]. The pink areas on upper left represent the three primary AOEs in south China.

Understanding the mechanisms shaping the present patterns of species diversity and endemism is fundamental in biogeography and evolutionary biology [13], . Operationally, an area that contains at least one unique species or a unique combination of species is an area of endemism (AOE) [21]. Biogeographic patterns of endemism in China have been studied for birds, reptiles, mammals, plants, insect, spiders and amphibians [22]–[28], leading to the identification of three congruent AOEs: the Southwest Mountainous Region (SMR, extending from the south of the Tibetan Plateau to the Yunnan Mountains); Taiwan and Hainan Island (figure 1). Of the three AOEs, Lei et al. [29] found the SMR have the highest richness of restricted range species and genera, but the highest richness of zone-restricted species is on Taiwan [30]. An AOE is a spatially and temporally bounded geographical area [21]. Species’ current distribution patterns might result from an amalgam of historical and current processes; the formation of endemic species is complicated and closely related to geology, climate, and the process of bio-evolution [29]. However, to date, relative to other spatial scales, the processes and mechanisms underlying the formation of areas of endemism remain poorly understood in China [31]–[32], in spite of its global status as one of the 17 megadiverse countries [33]–[34]. AOEs are determined by tectonism creating physical barriers and by biotic dynamics (dispersal, range expansions and contractions, and speciation as well as local persistence related to local stability) [21], [31]. Questions about AOEs may be best addressed by integrating phylogeographic analyses with ENMs, as better integration across geographical, geological and climate factors may form a more comprehensive model of endemism [35]–[38].

We selected the Red-headed Tree Babbler (*Stachyridopsis ruficeps*), a non-migrating bird in the Oriental realm [22], [24], to address the processes behind endemism in China. Currently, there are six recognized subspecies: *S. r. ruficeps*, *S. r. bhamoensis*, *S. r. davidi*, *S. r. praecognita*, *S. r. goodsoni* and *S. r. pagana* [39]–[40]. This species is primarily distributed in China, with peripheral or local populations in the adjacent eastern Himalayas (Nepal, Bhutan and India), northern Burma, Laos and Vietnam [39] (Fig. 1). This species inhabits broadleaf evergreen forest, bamboo stands and thick secondary bush growth in clearings from approximately 200–2500 m in China [40].

Studies of recent divergences are particularly attractive because the signatures of such events may not yet have been fully erased by time, and it can be more straightforward to infer processes from observed patterns of genetic variation [41]. The integration of multiple complementary approaches is a powerful way to understand the processes of diversification and speciation [7]. Therefore, in this paper, we attempt to address the principal drivers of the diversification process and the mechanisms underlying endemism in China by integrating a phylogeographic analysis of *S. ruficeps* with ENMs. Our results might also be able to add further insight into the species’ global distribution and endemism [42].

## Methods

### Ethics Statement

All of the samples used are unprotected bird specimens from the specimen collection of the National Zoological Museum, Institute of Zoology, Chinese Academy of Sciences (address: No1 Beichen West Road, Chaoyang District, Beijing, China). Birds were collected under a permit from the Forestry Department and conformed to the National Wildlife Conservation Law in China. No living animal experiments were conducted in the current research. These samples did not concern ethical issues. The Zoological Museum of the Institute of Zoology has the authority over sample collections and exemptions for sample exports/imports for scientific research purposes (No. 1999/84, provided by Article VII from CITES). See also the recent publication of Dai et al. 2011 in PLOS ONE [43].

### Sample Preparation

Blood or tissue samples were obtained from 179 *S. ruficeps* specimens collected from 16 sites in China including Taiwan (Fig. 1, Table 1). Additionally, three Nepalese specimens from the Natural History Museum of Denmark were used in this study. The samples were stored in 100% ethanol in the field and transferred to a −80°C freezer for long-term storage. Total genomic DNA was extracted from blood or tissue samples using the Qiagen™ extraction kit following the manufacturer’s instructions. Samples of *S. r. pagana* (only distributed in SC Vietnam) were not obtained despite our efforts, so the 478 bp cytochrome *b* (Cyt *b*) sequence acquired from GenBank (Access Number AF376886) was used to reconstruct the phylogenetic relationships among subspecies of *S. ruficeps* before the subsequent analysis.

**Table 1:** The map number, latitude, longitude and haplotypes of each sampling site.

Site label	Sampling site	Number	Latitude, longitude	Haplotypes identified	Subspecies
	Nepal	3	28.35N,84.23E	N1–N3	*S. r. ruficeps*
1	Chayu	4	28.56N, 97.08E	H1–H3	*S. r. ruficeps*
2	Yunnan	15	24.93N, 98.77E	H4–H15	*S. r. bhamoensis*
3	Panzhihua	7	27.04N, 101.97E	H16–H20	*S. r. bhamoensis*
4	Sichuan	12	30.07N,102.99E	H21–H29	*S. r. davidi*
5	Shaanxi	18	33.53N,107.83E	H30–H38	*S. r. davidi*
6	Hubei	12	31.57N,110.14E	H39–H47	*S. r. davidi*
7	Chishui	18	28.36N,105.94E	H33, H48–H61	*S. r. davidi*
8	Guiding	19	26.60N,107.15E	H33, H45, H53, H55, H62–H74	*S. r. davidi*
9	Guangxi	8	22.92N,106.48E	H75–H81	*S. r. davidi*
10	Guilin	15	25.20N,109.87E	H33, H55, HH82–H89	*S. r. davidi*
11	Guangdong	4	25.21N,113.60E	H90–H93	*S. r. davidi*
12	Hunan	5	28.79N,113.83E	H94–H98	*S. r. davidi*
13	Anhui	7	30.19N,118.55E	H99–H105	*S. r. davidi*
14	Fujian	9	26.57N,117.52E	H104–H112	*S. r. davidi*
15	Taiwan	13	23.46N,120.90E	H113–H123	*S. r. praecognita*
16	Hainan	13	18.99N,109.33E	H124–H135	*S. r. goodsoni*

### Polymerase Chain Reaction (PCR) and Sequencing

Two mitochondrial DNA (mtDNA) genes were amplified using the PCR; the 1340-bp cytochrome c oxidase I (COI) was amplified with the ‘universal’ primer pair L6615 and H7956 [44]. The 1104 bp Cyt *b* gene was amplified with the new specific primers CYTBUPA (5′-AAT ATA AYT TTA ATG GCT CTC AAT C-3′) and CYTBLOA (5′-ATA GTT TGA GTA TTT TGT TCT CTA-3′). The thermocycling program consisted of an initial denaturation at 94°C for 5 min, followed by 40 cycles of 94°C for 40 s, 47°C for COI and 52°C for Cyt *b* for 50 s, and 72°C for 1 min, plus a final extension at 72°C for 8 min. The same primers were used to sequence amplicons with a Big Dye Terminator Cycle Sequencing Kit v.2.0 run on an ABI 377 automatic sequencer. The sequences were assembled using Seqman II (DNASTAR) and visually proofread against the chromatograms. One sequence from *Macronous gularis* and two from *Stachyridopsis chrysaea* (amplified using the primers above) were used as outgroups. Three Nepalese specimens were amplified with the nested primers (see Table S1).

### Phylogenetic Analysis

The sequences were aligned using ClustalX [45], and haplotypes for Cyt b, COI and the combined sequence were generated in DnaSP 5.10 [46]. We concatenated the two mtDNA fragments into a combined dataset, and all further analyses were based on the combined dataset. Modeltest 3.07 [47] and the Akaike Information Criterion [48] were used to identify the appropriate nucleotide substitution models for phylogeny reconstruction. Maximum likelihood (ML) and Bayesian inference (BI) phylogenetic analyses were used to reconstruct the phylogenetic relationships among the haplotypes. We performed ML analyses in PHYML [49] and assessed nodal support using 1000 bootstrap replicates. BI was performed with MrBayes 3.12 [50] with the default parameters using the models selected by Modeltest. Initially, four Metropolis-coupled Monte Carlo Markov Chains (MCMCs) were run with trees sampled every 100 generations for 4 million generations or more until the standard deviation of split frequencies was below 0.01. The first 25% of generations were discarded as ‘burnin’, and the posterior probabilities were estimated for the remaining saved generations.

### Population Structures, Genetic Diversity and Gene Flow among Regional Groups

A hierarchical analysis of molecular variance (AMOVA) was performed to compare levels of genetic diversity within and among several possible population groupings of *S. ruficeps* using ARLEQUIN 3.1 [51] with 20,000 permutations. The groupings that maximized values of *F_CT_* and were statistically significant indicated the most parsimonious geographical subdivisions.

The numbers of haplotypes (*H*), values of haplotype diversity (*h*) and nucleotide diversity (π) for each regional group based on the result of the AMOVA were computed in ARLEQUIN 3.1 [51].

Genetic differentiation between regional groups was evaluated based on pairwise values of *F*_ST_. The statistical significance of the estimates was assessed after 10,000 permutations. Gene flow (*N*m) among groups was estimated according to the values of *F*_ST._ *F*_ST_ and *N*m were calculated using the software ARLEQUIN 3.1.

### Genetic Distance and Divergence Time Estimation

The net genetic distance (D) between geographical subdivisions was assessed by comparing the corrected average pairwise difference (PiXY – (PiX + PiY)/2) using MEGA 4.0 [52] under the Tamura-Nei substitution model [53] with a 500 replicate bootstrap. The PiXY is the average number of pairwise differences between two populations X and Y, and PiX and PiY are the average numbers of pairwise differences within each population. The divergence times among the geographical groups were estimated using the formula *t*_div time_ = D/2*µ*, where *µ* is the mutation rate of the combined dataset. Because no appropriate fossils were available with which to date the ancestor of Stachyridopsis, we were only able to use a conventional molecular clock, the avian mitochondrial gene (2%) [54]–[55], to provide an approximate estimation of the divergence time. Although the absolute timing of divergences may be debatable, the sequence of events and the relative timing depicted here are expected to approximate the evolutionary history of *S. ruficeps*.

### Population Demographic History

Values of Tajima’s *D* [56] and Fu’s *Fs* [57] were used to assess the evidence for population expansion for the geographical groups arranged by AMOVA partitions and phylogenetic topology. We also used Bayesian skyline plots (BSP) [58] implemented in the software program BEAST 1.4.7 [59] to depict the dynamics of population size dating back to the time of the most recent common ancestor (TMRCA). We performed BSP for each geographical group and all groups combined. All analyses were run for 100 million iterations, sampling genealogy and population size parameters every 2000 iterations and discarding the first 10% as burn-in. The nucleotide substitution model we used was TVM+I+G, as selected in Modeltest [47]. Although the mean substitution rate was fixed by assuming a conventional avian molecular clock (see Results section), we used an uncorrelated lognormal model [60] to account for rate variation among lineages. Default settings of Bayesian priors were used. In addition, the TMRCA of each geographical group and all groups combined were estimated using the same mutation rate as above. The results were summarized using TRACER 1.3 [61].

### Ecological Niche Modeling

Species’ ecological characteristics are generally conserved over moderate periods of time despite profound changes in climatic and environmental conditions [16], [62]–[64]. As there were no earlier records of environmental conditions during the Pleistocene glacial cycle available for China, even though the divergence times among lineages were prior to the last glacial maximum (LGM, 21,000 yr BP)(see below), we performed ENM to estimate the potential distributions for *S. ruficeps* in the present and during LGM, with the goal of modeling the impacts of Pleistocene climatic oscillations on the species’ distribution. We modeled the predicted suitable habitat using maximum entropy methods in the program MAXENT 3.3.2 [65], which has been shown to be robust for variable sample sizes and to perform well compared with other methods in predicting past and present species distributions [66]–[68]. We considered the 19 bioclimatic variables at a 2.5′ spatial resolution available from the WorldClim database (see below) [69]. LGM climate data were simulated from two models: the Community Climate System Model (CCSM) [70] and the Model for Interdisciplinary Research on Climate (MIROC) [71]. To minimize model over-fitting, we calculate Pearson’s correlation coefficient (*r*) between each pair of variables using R. Variables with *r* >0.8 were considered as highly correlated, and we selectively removed one variable from each of these pairs. We chose variables that represent climate seasonality or extremes rather than average temperature or precipitation. The final model included 9 variables: BIO2–mean diurnal temperature range; BIO3–isothermality; BIO5–max temperature of warmest month; BIO6–min temperature of coldest month; BIO7–annual temperature range; BIO8–mean temperature of the wettest quarter; BIO13–precipitation in the wettest month; BIO14–precipitation in the driest month and BIO15–precipitation seasonality. Species occurrence data included the 82 sampling sites recorded in the field and sightings from some bird-watching sites (downloaded from http://birdtalker.net/index.asp) with georeferenced data specific enough for the longitude and latitude to be estimated with confidence using Google Earth (http://www.google.com/earth). A total of 220 presence records were used after removing the localities that were separated from each other by less than 0.1 geographical degrees to minimize spatial autocorrelation.

The ENM was constructed based on current bioclimatic variables, then projected to the LGM variables built on the CCSM and MIROC models. The output map was generated by averaging the suitable probability within each grid cell. This approach is considered advantageous because it is not biased by limited absence records [66], although it does assume that preferences for climatic conditions do not change over time. We used the default convergence threshold (10^−5^) and set the maximum iterations to 2000 and number of replicates to 10. The logistic output format was chosen, which produces continuous probability values for each grid cell from 0 to 1, an indicator of the relative suitability for the species. Twenty-five percent of the localities were randomly selected to train the model and the remaining 75% to test the model performance. We also performed jackknife resampling to measure variable importance and explore the primary environmental factors restricting the Red-headed Tree Babbler’s geographic distribution. Model performances were evaluated by averaging the area under the curve (AUC) values for the receiver operating characteristic (ROC) curves over ten replicate runs. An AUC >0.5 indicates that a model performs better than random, and an AUC >0.9 indicates an excellent performance [72].

**Figure 2:**
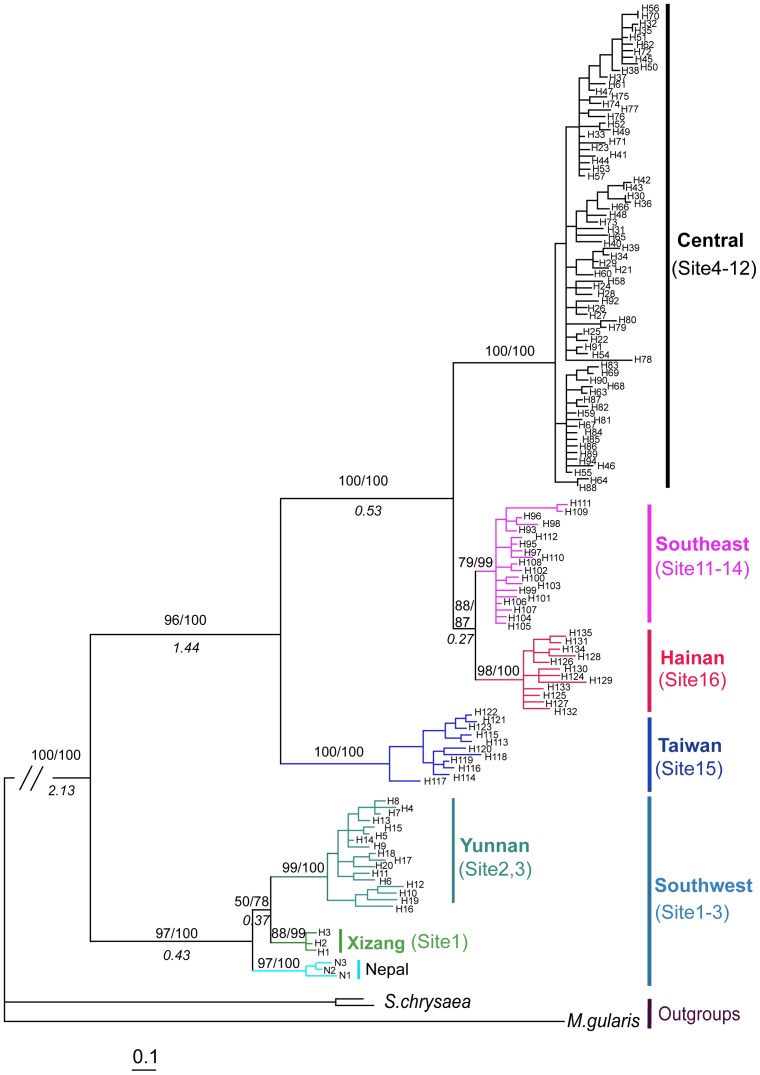
Bayesian trees of all combined mitochondrial haplotypes. Values above branches represent maximum likelihood bootstrap values and Bayesian a posteriori probability; values below branches represent the divergence time between lineage groups. Outgroups names are shown. The site numbers and haplotype names correspond to Table 1.

To assess the impacts of the ecological niche on the formation and maintenance of separate lineages, we also modeled the suitable habitats for the inferred lineages. We built reduced-ENM models based only on the localities in the three AOEs (proven to be three monophyletic lineages; Southwest, Taiwan and Hainan, see Figs. 1 and 2) and the remaining sites, including the lineages of Central and Southeast. To facilitate model interpretation, we selected the widely used lowest presence threshold (LPT) [73] to distinguish ‘suitable’ from ‘unsuitable’ areas.

### Niche Similarity

Niche similarity between adjacent lineages was calculated following Warren et al. [74], who propose two metrics of niche overlap based on ENM predictions, namely Schoener’s D [75] and ‘Warren *et al.’s’ I* [74]. These statistics quantify niche overlap and range from 0 (no overlap) to 1 (complete overlap). First, niche-overlap values were calculated from the ENMs for pair of populations using ENMtools [74]. To test the null hypothesis that the niches of two populations are identical, we performed the identity test in ENMtools, which evaluates equivalency between ENMs by comparing the observed values of *D* and *I* for the two models with a distribution of values of *D* and *I* based on randomized pseudoreplicates. This distribution is generated by randomly assigning occurrence points from both groups into one lineage or the other, simulating the potential overlap of a group of points occurring across a given geographical area [74]. As we are primarily reporting interactions between sister lineages, we did not employ the possible phylogenetic corrections for these analyses [74]. We calculated the observed *D* and *I* values and simulated distributions of *D* and *I* using 100 pseudoreplicates for all pairwise comparisons of the inferred lineages. We also wish to determine whether ENMs were more similar than expected by chance based on the geographical regions in which they reside. We used the background randomization procedure in ENMtools, which compares the observed niche overlap values to a null distribution of 100 overlap values generated by comparing the ENM of one taxon to an ENM created from random points drawn from the geographic range of the other taxon [74]. Because this process is then repeated for both taxa in the comparison, two null distributions were generated per analysis.

## Results

### Phylogenetic Analysis

We obtained 938 bp of the partial Cyt *b* gene and 1237 bp of the partial COI gene from 179 individuals collected in China. The Cyt *b* sequences contained 132 polymorphic sites, defining 104 haplotypes (GenBank Access Number HM191271–HM191346, HQ917474–HQ917501). The COI sequences yielded 167 polymorphic sites, identifying 94 haplotypes (GenBank Access Number HM191347–HM191416, HQ917502–HQ917525). The combined dataset identified 135 haplotypes (Table 1), and each of the Nepal samples identified a unique haplotype.

The results from Modeltest indicated that the best model for the combined dataset was TVM+I+G (I = 0.7045, G = 1.9012). Phylogenetic reconstructions of the ML and BI analyses produced nearly identical topologies that broadly corresponded to distinct geographical regions (Fig. 2). Five major well-supported clades were identified that divided *S. ruficeps* into the Southwest (sites1–3), Taiwan (site 15), Hainan (site 16), Southeast (sites 11–14) and Central (sites 4–12) (Figs 1 and 2, Table 1). The relationships among these lineages were fully supported (bootstrap>75%), of which the Southwest, Taiwan and Hainan phylogroups were coincident with the three primary AOEs; the Southwest phylogroup constituted a basal lineage, whereas the Hainan phylogroup was represented as a tip clade. These clades are allopatric with the exception of two sympatric sites on Guangdong and Hunan (sites 11 and 12), which may be a secondary contact zone between the Southeast and Central clades. Similar subdivisions have also been evident in other bird species [76]. Most of the locations of the geographic phylogroups were consistent with the subspecies distribution ranges except for the subspecies *S. r. davidi*, which included two monophyletic groups (Southeast and Central) (Fig. 1, Table 1). One Cyt *b* sequence of *S. r. pagana* from Vietnam (AF376886, 477 bp sequence available but not included in Fig. 2) was nested within the Central clade. The Southwest phylogroup was closely related to the Nepal samples of *S. r. ruficeps*, which could also be divided into two subclades (Figs. 1 and 2, Table 1).

In the AMOVA, the highest amount of genetic variance between groups (*F_CT_* = 0.89, p<0.001) was found when we subdivided the samples into six groups based on the phylogenetic results (Table S2). A long-term absence of gene flow among all geographical groups was indicated by the significant, high *F_ST_* and the negligible *Nm* (Table 2). The haplotype diversities of the geographical groups ranged from 0.833 to 0.99, and nucleotide diversities ranged from 0.00046 to 0.00485 (Table 3).

**Table 2:** Pairwise population differentiation and gene flow among populations (*N*m) of the five lineages based on mtDNA haplotype frequencies.

Groups	Xizang	Yunnan	Taiwan	Hainan	Southeast	Central
**Xizang**		0.35	0.04	0.04	0.03	0.04
**Yunnan**	0.59^***^		0.06	0.05	0.04	0.04
**Taiwan**	0.93^***^	0.90^***^		0.07	0.06	0.06
**Hainan**	0.93^***^	0.91^***^	0.88^***^		0.29	0.14
**Southeast**	0.95^***^	0.92^***^	0.89^***^	0.63^***^		0.15
**Central**	0.93^***^	0.93^***^	0.90^***^	0.79^***^	0.77^***^	

Note: Below diagonal: *F*-statistics for pairwise population differentiation, *** *P*<0.001 after 10000 permutations. Above diagonal: Nm among populations.

**Table 3:** Number of samples (**N**), haplotypes (**H**), haplotype diversity (**Hd**), nucleotide diversity (**π**), Tajima’s *D*, Fu's *Fs* and TMRCA of the six geographic groups of *Stachyridopsis ruficeps.*

Groups	N	H	Hd	π	*D*	*F*s	TMRCA(mya)
**Xizang**	4	3	0.833	0.00046	−0.71	−0.887	
**Yunnan**	23	18	0.976	0.00485	−1.51	−4.48	0.369
**Taiwan**	13	11	0.974	0.00376	−1.305	−2.666	0.389
**Hainan**	13	12	0.987	0.00374	−1.563	−4.319*	0.249
**Southeast**	21	19	0.99	0.00266	−1.78	−12.781***	0.215
**Central**	106	73	0.988	0.00338	−2.18**	−76.446**	0.303

* *P<*0.05, **P<0.01, ***P<0.001.

### Genetic Distance and Divergence Time

The net genetic distance between the Southwest group and the remaining clades was 0.042 (0.0372–0.0454), so the basal split time was estimated to be 2.13 Ma (1.86–2.27 Ma); the next oldest basal clade was Taiwan, with a net genetic distance of 0.0288 (0.0252–0.0332) and a divergence time of 1.44 Ma (1.26–1.66 Ma). The net genetic distance between Xizang and Yunnan was 0.0074 (0.005–0.009), and the divergence time was 0.37 Ma (0.25–0.45 Ma). The Hainan and Southeast lineages diverged most recently, with a net genetic distance of only 0.0054 (0.004–0.006), and a divergence time of 0.27 Ma (0.2–0.3 Ma), more recent than the divergence between these groups and Central clade of *S. r. davidi* at approximately 0.53 Ma (0.45–0.66 Ma) (Fig. 2). The estimated net distances and divergence times among the phylogroups are shown in Table S3.

### Population Demographic History

**Figure 3:**
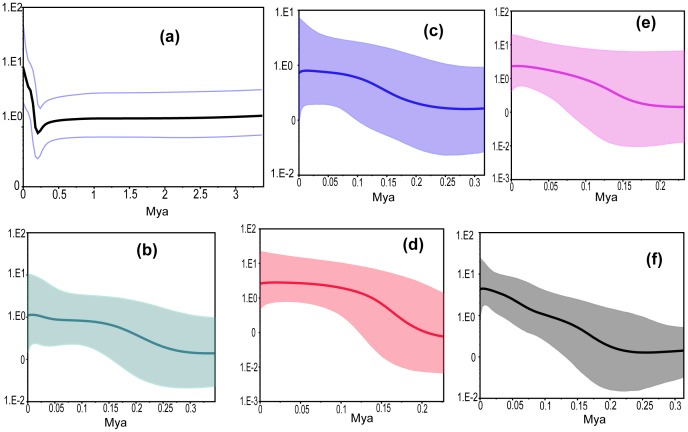
Bayesian skyline plots of past population demographic trends in mitochondrial lineages. x-axis time in 10^6^ yr BP; y-axis is estimated population size [units = *Neτ* (the product of effective population size and the generation time in years). The mean estimates are joined by a solid line, and dashed lines delineate the 95% HPD limits. (a) All sequence; (b) Southwest; (c) Taiwan; (d) Hainan Island; (e)Southeast; (f) Central.

Following the phylogenetic tree results, the groups defined for demographic expansion tests included the four clades and the two subclades (Fig. 2; Table 3). Negative values of Fu's *Fs* and Tajima’s *D* were found for all the six groups, although only the Hainan, Southeast and Central phylogroups were statistically significant (*F_S_* α = 0.02; *D* α = 0.05). The BSP simulated the changes in population size since the TMRCA (Fig. 3). For the whole dataset, the TMRCA was dated to 3.299 Ma (95% CI: 1.935–4.784). Due to its small sample size, the Xizang group (n = 4) was excluded from the BSP analysis. Recent population increases were observed for all five of the other groups, with population growth since 0.25, 0.25, 0.25, 0.15 and 0.20 Ma for the Yunnan, Taiwan, Hainan, Southeast and Central groups respectively. TMRCAs were inferred back to 0.369 Ma (95% CI: 0.199–0.570), 0.389 Ma (95% CI: 0.146–0.626), 0.249 Ma (95% CI: 0.139–0.379), 0.215 Ma (95% CI: 0.087–0.401) and 0.303 Ma (95% CI: 0.155–0.517) for the Yunnan, Taiwan, Hainan, Southeast and Central groups, respectively (Table 3).

### Ecological Niche Modeling and Equivalency

**Figure 4:**
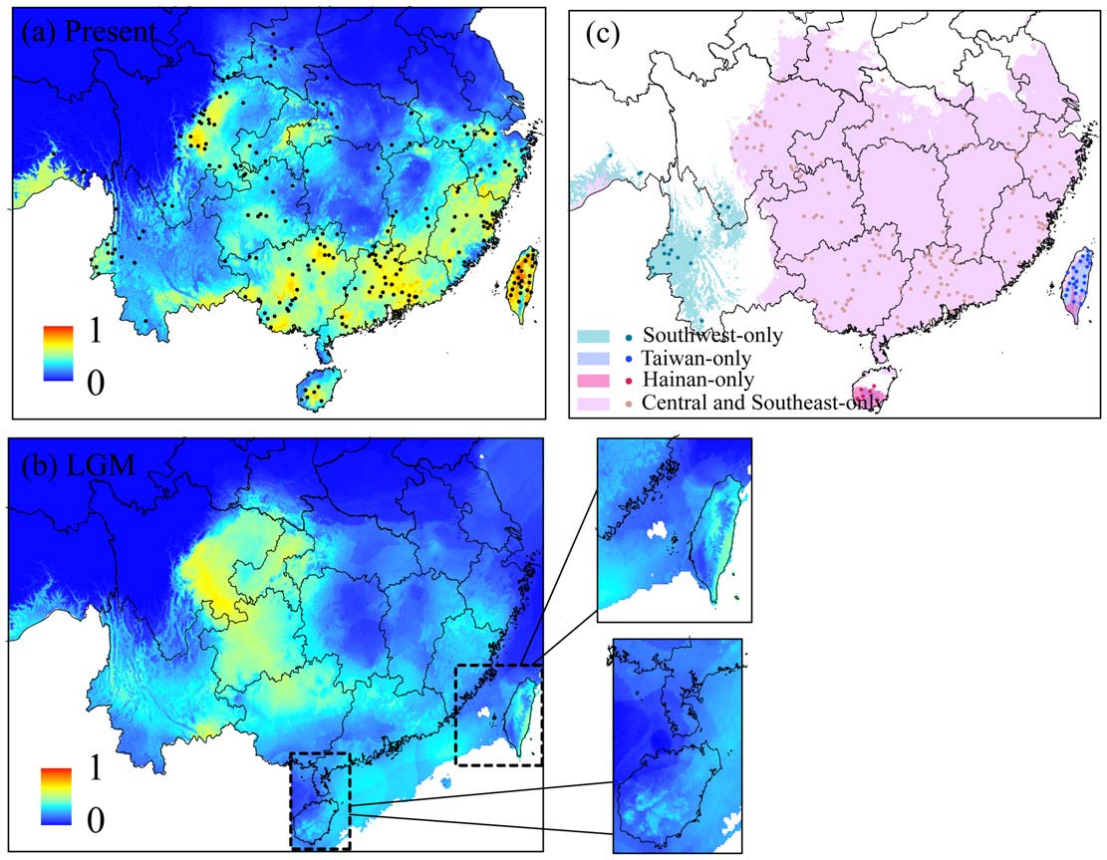
The spatial distributions of *S. ruficeps* predicted by Maxent. Present (a) and the LGM (b) using all localities, and the reduced distributions for each clade (c) in the present. “·” designates the observed distribution of *S. ruficeps*. Levels of shading represent continuous logistic probabilities of bioclimatic suitability, ranging from highest suitability (red) to unsuitable (blue) habitat for the full distribution. For the reduced distributions, we only used two states suitability or unsuitability.

MAXENT appeared to perform well for the full ENM, with an average training AUC of 0.965±0.002. These binomial probabilities (p<<0.0001) for every run suggested that the model predicted significantly better than random expectations at all thresholds. The present-day spatial prediction generated for the full ENM was largely congruent with the known species distribution (Fig. 4a), and the species’ suitability in the three AOEs was lower during the LGM than in the present. Although there were land bridges over the Taiwan and Qiongzhou Straits during the LGM, both areas appeared unsuitable at the LGM (Fig. 4b).

Both the present-day and LGM predictions were consistent with the findings of our molecular analyses. The model predicted that populations of Red-headed Tree Babbler were separated by climatically unsuitable habitats (Fig. 4a). This result corroborated the analyses of population structure, which suggested that there were strong barriers to dispersal. In addition, both the geographic extent and relative suitability of habitats are predicted to have been reduced at the LGM in comparison to the present (Fig. 4b). The latter result paralleled the demographic analyses, which suggested that populations might have increased in size in response to a geographic expansion of suitable habitats.

The reduced ENMs developed using localities from clade A (training AUC values 0.995±0.001), clade B (0.998±0.001), clade C (0.998±0.001) and clade D+E (0.960±0.004) alone also performed well in predicting the range-wide distribution of *S. ruficeps*. The predicted distributions for each lineage closely matched their present distributions (Fig. 4c). The variables with the greatest contributions to the models for each lineage were as follows: BIO3 (isothermality) contributed most to Southwest (62.9%), BIO7 (temperature annual range) contributed most to Taiwan (77.8%) and Hainan (76.5%), and BIO2 (mean diurnal temperature range) contributed most to Southeast and Central (57.5%).

**Figure 5:**
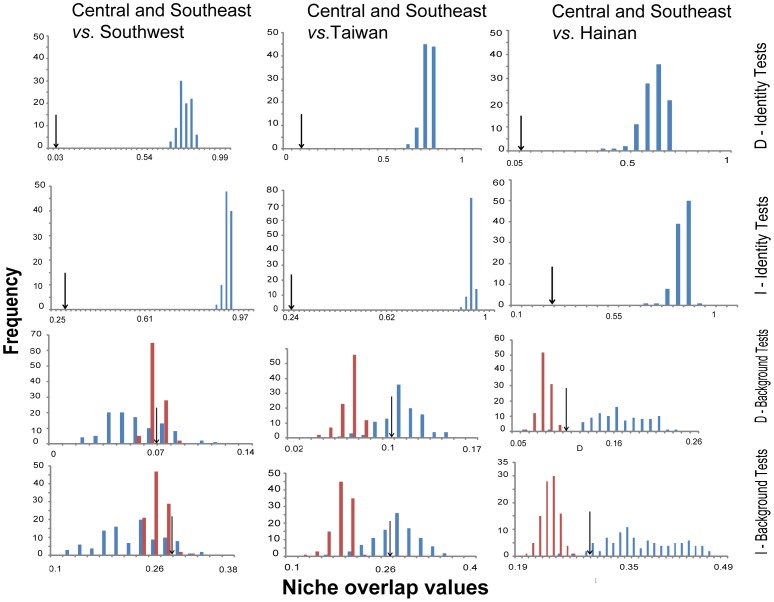
Sampling for the niche similarity test. *I* and *D* are two distinct measures of niche similarity (Warren et al., 2008). The arrows indicate the observed values relative to the frequency distributions of random replicates from the niche-identity and niche-background tests.

The similarity tests are presented in Table 4 and Figure 5. All of the identity tests showed that Schoener's *D* and *I* values for the pairwise comparisons of interest were significantly lower than expected from a random distribution for all comparisons (*P*≤0.01), so the null hypothesis of niche identity for all adjacent lineages was rejected. Thus, the niches of all the lineages are not identical to each other. The null hypothesis of background test could not be rejected for either direction of the Southwest vs. Central and Southeast lineage (Table 4, *P*>0.05). This indicates that the niches are only as similar as can be expected from random niches drawn from the available climates, so the ecological niches may have diverged between these two lineages. For each island lineage compared with the Central and Southeast lineage, the null hypothesis of background test was rejected for one direction (Table 4, *P*≤0.01), indicating that the niches are more similar to each other than expected at random [74], which is evidence for strong niche conservatism. However, in terms of the very significantly differentiated niche identity (Table 4, *P*≤0.01), although the niches of the island lineages vs. Central and Southeast lineage were significantly similar, they are not identical [16].

**Table 4:** Tests of niche similarity. Each test followed by an assessment of statistical significance.

Lineage	Identity test		Background test	
	D	I	D	I
**Southwest vs. Central and Southeast**	0.079**	0.289**	0.079 ns, ns	0.289 ns, ns
**Taiwan vs. Central and Southeast**	0.107**	0.268**	0.107**, ns	0.268**, ns
**Hainan vs. Central and Southeast**	0.111**	0.299**	0.111**, ns	0.299**, ns

Significant of background tests are given as “other lineage predicting Central and Southeast lineage, Central and Southeast lineage predicting other lineage”.

* , *P*≤0.05,

** , *P*≤0.01,

ns, *P*>0.05.

## Discussion

### Phylogeographic Structure and Lineage Endemicity

Our analysis revealed that the haplotype lineages of *S. ruficeps* are exclusive to geographic regions and that each AOE harbors a unique monophyletic clade: Southwest clade in SMR, Taiwan clade in Taiwan and Hainan clade in Hainan Island. The Southwest clade was the basal clade in our study, followed by the Taiwan clade, whereas Hainan was the proximal clade. Comparisons of the genetic patterns of co-distributed species may reveal historical processes that have occurred at the landscape scale. A congruent pattern was found in *Alcippe morrisonia,* which is distributed in similar geographical areas and habitats [76], although the TMRCA and divergence times of the main lineages were approximately twice those of *S. ruficeps* (the molecular clock, effective population size, generation time or the species’ evolutionary history may cause this difference). Congruent patterns are also evident in other birds, such as *Leucodioptron canorum* [77] and *Aegithalos concinnus* [78], despite their relatively restricted distribution ranges. This congruent pattern of lineage divergence may be the result of similar responses to physiographic and environmental shifts during the late Pliocene and Pleistocene. Deep isolated lineages with disjunctive geographical ranges, negligible *Nm*, and the significant ecological niche divergence led us to regard the populations in the three AOEs as potential distinct species. Further studies of their diagnosability and vocalizations and nuclear data for modelling gene flow are required for a full assessment of the taxonomic ranks of these populations. The endemic lineages may have independently undergone long-term evolution and adaption to local environments, which implies that some form of isolating mechanisms have evolved.

### Vicariance Hypothesis

The disjunctive distribution of the phylogroups indicated that an allopatric process may be the most likely mode of divergence, and geological events might be important factors for this geographic isolation. The initial divergence of the species divided the Southwest lineage from the others at the Qionglai Mts and Ta-liang Mts. approximately 1.86–2.27 Ma. This geographical divide has been documented in numerous species of plants and animals [43], [76], [78]–[79]. The uplift of the Tibetan Plateau had profound effects on the geological environment of the Plateau and adjacent areas [80] and may have promoted the habitat fragmentation of species [81]. Although the timing of the tectonic uplift of the Tibetan Plateau remains controversial [82]–[83], the strongest uplift, involving the whole Plateau and its marginal mountains, commenced at 3.6 Ma, after which there were two additional tectonic uplifts [84].

Considering the divergence time and the middle or lower altitude distribution of this species, we hypothesized that the uplift of the Tibetan Plateau might be an important factor in the phylogeographic breaks within *S. ruficeps*. The climatic fluctuations during the Pliocene/Pleistocene boundary might also have been an important cause of the isolation of the Southwest lineage. From 2.4 Ma onward, ice sheets began to expand in the Northern Hemisphere [85], resulting in altitudinal shifts [86] and contractions of species distributions. The importance of Pliocene/Pleistocene boundary climate fluctuations for avian speciation has also been supported by most of the North American birds [87]. Our ENMs (Figs. 4a, b) suggest that the Qionglai Mts and Ta-liang Mts. (fig. 1) were less suitable during both the glacial (LGM) and interglacial (present day) stages. Therefore, we could associate the initial isolation with this cooling event, the topographic barriers as a primary cause of the isolation of *S. ruficeps* populations dating at least from the LGM, and the absence of gene flow between these lineages, which led to incipient allopatric diversification (Fig. 1).

The divergence of the Taiwan group occurred approximately 1.26–1.66 Ma. A long independent evolution of the lineages inhabiting this island has also been found in other birds [76]–[77]. Pleistocene glacial-interglacial cycles were likely to have resulted in the repeated isolation and divergence of haplotypes on islands with favorable habitats [78], [88]. For birds with poor dispersal ability, the Taiwan Strait (Fig. 1) might have been an important barrier during interglacial periods when the land bridge disappeared. Similar to our Cyt *b* result, Li *et al*. [89] reported completely interrupted gene flow between Hwamei and Taiwan Hwamei (*L. canorum* and *L. taewanum*) before 0.5 Ma. We may wonder why the independent evolution of the Taiwan population could be sustained over such a long period, as Taiwan was repeatedly connected and disconnected from the East Asian continent during the Pleistocene [90]. Exogenous factors, such as habitat barriers, may have contributed significantly to maintaining the evolutionary isolation. During the LGM, although the island connected with the mainland, ENM showed low suitability for the land-bridge areas (Fig. 4b). The reconstructed paleo-vegetation of East Asia also suggests that the Taiwan Strait was covered by savanna rather than evergreen broad-leaved forest during glacial periods [91]–[92]. Therefore, we assume that the species was unlikely to survive in these areas during the LGM. The absence of appropriate habitat may have constricted gene flow between the island and mainland populations despite the presence of a land bridge.

Similar to the Taiwan lineage, the Hainan Island lineage may also have been isolated by the Qiongzhou Strait or unsuitable habitat. However, the Hainan population diverged from the mainland population only during the period of the most violent climatic cycles in the middle Pleistocene [93], which was much more recently than the Taiwan population. Our result is in agreement with previous studies, such as those of *A. morrisonia* [94] and *L. canorum* [77], the results of which showed that the divergence time of Hainan lineages from the mainland lineages is more recent than that of Taiwan lineages from the mainland lineages. Generally, Taiwan has a greater number of endemic species than Hainan [29]–[30], which can be considered in relation to its isolation time, elevation (with more suitable montane habitats in Taiwan than in Hainan) [90], [94] and remote isolated distance (230 km from the mainland, compared to 20 km in the case of Hainan) [94]. Our result supports the conclusion that ecological barriers might be the most plausible explanation for the different degrees of divergence between the two islands and China’s mainland, which is in agreement with general island theory [95].

### Pleistocene Refugia Hypothesis

The patterns of endemism observed today might be a relict pattern maintained by periodic eliminations from large areas with the exception of areas that remained stable during the upper Pleistocene due to local topographic moderation of the climate [96] and because species can easily track climatic shifts within steep montane habitats [31]. The divergence of lineages within *S. ruficeps* occurred approximately 0.267–2.27 Ma. Although these estimates are associated with significant uncertainty,they all fall within the Pleistocene. Isolated refugia over one to several full glacial cycles could induce speciation [97]–[99]. Even without niche distributions earlier than the LGM, the BSP results showed that each lineage had undergone population expansion after the initial isolation. Compared with other species in southern China, such as *Taxus wallichiana* [78], *L. canorum* [100], *Dysosma versipellis* [79], *A. morrisonia* [76], and *Bambusicola thoracica thoracica* [101], congruence among these genetic structures across these subregions support a long-term restriction of southern China to multiple independent localized refugial areas, allowing the populations in these areas to persist through several climatic cycles in heterogeneous landscapes. Quaternary refugial isolation was also likely to have enhanced allopatric (incipient) species formation in temperate plants in East Asia [79], [92], [102]–[104].

Both in the present and during the LGM, past climatic cycles may have profound impacts on the genetic variability and distribution of endemic lineages. During the LGM, there was more suitable habitat for the Southwest than for the island lineages, although populations might also have contracted to the western Chinese boundary region or the Himalayas (Fig. 4b); the genetic results showed further phylogeographic structuring and greater genetic variation. Mountainous areas may play a key role in speciation [105], as they create a mosaic of microclimates of relative stability that allow species to persist over much of their range [102]. The SMR has the most heterogeneity and biogeographical complexity in China. Considering the “ecological island” effect in the SMR [30], genetic exchange was restricted during climate oscillations. Our results confirmed the importance of mountainous environments as barriers in preventing gene flow, promoting speciation and maintaining high endemism [28].

### Ecological Adaptation Hypothesis

Once populations have become genetically differentiated, their divergence status can be maintained if they have differentially adapted to regional ecological conditions, as geographic differences in selection pressures can act as a strong barrier to gene flow [106]–[107].

Even with the same suite of environmental conditions available to them, the lineages’ tolerance of the environmental conditions could diverge significantly. Our results predict almost complete ecological separation between all adjacent lineages (Fig. 4, 5). This suggests that environmental preferences are labile even over recent timescales, and species may evolve significant differences even between recently diverged lineage pairs as natural selection acts on populations in ecologically heterogeneous environments [108]. Niche divergence may lead to lineage formation when populations adapt to new environments [108].

Overall, the formally recognized subspecies of *S. ruficeps* can mostly be confirmed genetically as distinct phylogeographic units; not only have these units diverged in allopatry, but they also show distinctive adaptation trends, Thus, each phylogroup might have undergone divergent evolution in physiological and/or life history traits, with adaptation to different eco-climatic conditions. Taiwan has a subtropical island climate (warm and humid all year round). Hainan Island has a tropical monsoon maritime climate (minimal temperature annual range, with distinct dry and rainy seasons). The southwest mountain region is affected by the Indian monsoon (with a rainy summer and autumn) and, thus, has a relatively drier climate. The Central/Southeast lineages are exposed to the Pacific monsoon and have a cold winter and warm/humid summer [109].

This climatic heterogeneity should have ecologically constrained the potential for postglacial expansions and then prevented effective migrations among ecologically distinct regions. Therefore, the current pattern of distribution of the three AOE groups in China appears to be defined by adaptive differences reinforcing the role of physical barriers. As a consequence, there has been little or no gene flow and the patterns of differentiation created during historical isolation have therefore been maintained. Thus, our study illustrated that the lineages representing separate areas of endemism have a long history of independent evolution, enabling adaptations to local conditions. Speciation across geographical barriers can be influenced by niche divergence in ecologically distinct habitats [3], [4], [6]. The highly diversified habitats and geographically separated environments might have reinforced the isolation of populations in maintaining the genetic lineage or species endemism.

### Conclusion

Intraspecific data are rarely used to illustrate endemism. In this study, we integrated the phylogeography of the non-migrating oriental bird *Stachyridopsis ruficeps* and ENMs to address the principal drivers of avian diversification and the formation of endemism in China. We found evidence from both the mitochondrial DNA and the modeled distribution of the species that there is significant geographic structure in *S. ruficeps*. Deeply isolated endemic lineages with disjunctive geographical ranges were generally separated before the climatically most unstable Late Pleistocene. The phylogeographic patterns of our study indicate that vicariant events due to geographical or ecological barriers might be the drivers or facilitators in forming these endemic lineages or putative species, after which ecological niche differentiation resulted in a situation where expanding populations remained parapatric. Refugia are directly responsible for maintaining the endemic lineages, which may supply the source for speciation. Major biotic responses to climatic change involve persistence and resilience rather than large-scale migration, indicating the importance of dynamic evolutionary processes and a mosaic of habitats in heterogeneous landscapes for the persistence of species through changing environmental conditions. The deep isolation and complex genetic differentiation of the study species highlight the SMR as the center of origin for genera and species. However, as a longer-isolated and more distant island, Taiwan has the highest proportion of strict endemics.

## Supporting Information

Table S1 **The nested primers and thermocycling program for the three Nepal specimens.**
(DOC)

Table S2 **Results of the hierarchical analyses of genetic variance (AMOVA).**
(DOC)

Table S3 **Corrected mean lineage distances and divergence times of the main lineages and the genetic variation within each lineage.**
(DOC)

## References

[1] Brown JH, Stevens GC, Kaufman DM (1996) The geographic range: size, shape, boundaries, and internal structure. Annual Review of Ecology and Systematics 27: 597–623.

[2] Gaston KJ (2003) The Structure and Dynamics of Geographic Ranges. Oxford University Press, Oxford.

[3] Graham CH, Ron SR, Santos JC, Schneider CJ, Moritz C (2004) Integrating phylogenetics and environmental niche models to explore speciation mechanisms in dendrobatid frogs. Evolution 58: 1781–1793. 15446430 10.1111/j.0014-3820.2004.tb00461.x

[4] Raxworthy CJ, Ingram CM, Rabibisoa N, Pearson RG (2007) Applications of Ecological Niche Modeling for Species Delimitation: A Review and Empirical Evaluation Using Day Geckos (*Phelsuma*) from Madagascar. Systematic Biology 56: 907–923. 18066927 10.1080/10635150701775111

[5] Raxworthy CJ, Martínez-Meyer E, Horning N, Nussbaum RA, Schneider GE, et al (2003) Predicting distributions of known and unknown reptile species in Madagascar. Nature 426: 837–841. 14685238 10.1038/nature02205

[6] Rissler LJ, Apodaca JJ (2007) Adding more ecology into species delimitation: ecological niche models and phylogeography help define cryptic species in the black salamander (*Aneides flavipunctatus*). Systematic Biology 56: 924–942. 18066928 10.1080/10635150701703063

[7] Lozier JD, Mills NJ (2009) Ecological Niche Models and Coalescent analysis of gene flow support recent allopatric isolation of Parasitoid Wasp populations in the Mediterranean. PLoS ONE 4: e5901. 19521534 10.1371/journal.pone.0005901 PMC2691581

[8] Palsbøll PJ, Bérubé M, Allendorf FW (2007) Identification of management units using population genetic data. Trends in Ecology and Evolution 22: 11–16. 16982114 10.1016/j.tree.2006.09.003

[9] Bowie RCK, Fjeldså J, Hackett SJ, Crowe TM (2004) Speciation in space and time: mtDNA phylogeography of Olive Sunbirds (Nectarinia olivacea/obscura) across the African continent. Molecular Phylogenetics and Evolution 33: 56–74. 15324839 10.1016/j.ympev.2004.04.013

[10] Bickford D, Lohman DJ, Sodhi NS, Ng PKL, Meier R, et al (2007) Cryptic species: a new window on diversity and conservation. Trends in Ecology and Evolution 22: 148–155. 17129636 10.1016/j.tree.2006.11.004

[11] Balke M, Ribera I, Hendrich L, Miller MA, Sagata K, et al (2009) New Guinea highland origin of a widespread arthropod super tramp. Proceedings of the Royal Society B: Biological Sciences 276: 2359–2367. 19364747 10.1098/rspb.2009.0015 PMC2690458

[12] Lohman DJ, Ingram KK, Prawiradilaga DM, Winker K, Sheldon FH, et al (2010) Cryptic genetic diversity in “widespread” Southeast Asian bird species suggests that Philippine avian endemism is gravely underestimated. Biological Conservation 143: 1885–1890.

[13] Wiens JJ, Donoghue MJ (2004) Historical biogeography, ecology and species richness. Trends in Ecology and Evolution 19: 639–644. 16701326 10.1016/j.tree.2004.09.011

[14] Kozak KH, Graham CH, Wiens JJ (2008) Integrating GIS-based environmental data into evolutionary biology. Trends in Ecology and Evolution 23: 141–148. 18291557 10.1016/j.tree.2008.02.001

[15] Wiens JJ, Graham CH (2005) Niche conservatism: integrating evolution, ecology, and conservation biology. Annual Review of Ecology and Systematics 36: 519–539.

[16] Peterson AT (2011) Ecological niche conservatism:a time-structured review of evidence. Journal of Biogeography 38: 817–827.

[17] Peterson AT (2001) Predicting species’ geographic distributions based on ecological niche modeling. Condor 103: 599–605.

[18] Guisan A, Thuiller W (2005) Predicting species distribution: offering more than simple habitat models. Ecology Letters 8: 993–1009. 10.1111/j.1461-0248.2005.00792.x 34517687

[19] Jakob SS, Ihlow A, Blattner FR (2007) Combined ecological niche modelling and molecular phylogeography revealed the evolutionary history of Hordeum marinum (Poaceae)–niche differentiation, loss of genetic diversity, and speciation in Mediterranean Quaternary refugia. Molecular Ecology 16: 1713–1727. 17402985 10.1111/j.1365-294X.2007.03228.x

[20] Moritz C, Patton JL, Schneider CJ, Smith TB (2000) Diversification of rainforest faunas: An integrated molecular approach. Annual Review of Ecology and Systematics 31: 533–563.

[21] Crother BI, Murray CM (2011) Ontology of areas of endemism. Journal of Biogeography 38: 1009–1015.

[22] Zheng ZX, Zhang RZ (1959) Delimitation of Zoogeographic Regions and Insect Zoogeographic Regions of China. Science Press, Beijing.

[23] Stattersfield AJ, Crosby MJ, Long AJ, Wege DC (1998) Endemic Bird Areas of the World. Priorities for Biodiversity Conservation. BirdLife International, Cambridge, U.K.

[24] Zhang RZ (1999) Zoogeographic Regions of China. Science Press, Beijing.

[25] Xie Y, John M, Li DM (2004) Study on biogeographical divisions of China. Biodiversity and Conservation 13: 1391–1417.

[26] Chen YH, Bi JF (2007) Biogeography and hotspots of amphibian species of China: implications to reserve selection and conservation. Current Science 92: 480–489.

[27] Meng K, Li SQ, Murphy RW (2008) Biogeographical patterns of Chinese spiders (Arachnida: Araneae) based on a parsimony analysis of endemicity. Journal of Biogeography 35: 1241–1249.

[28] Huang XL, Qiao GX, Lei FM (2010) Use of Parsimony Analysis to Identify Areas of Endemism of Chinese Birds: Implications for Conservation and Biogeography. International Journal of Molecular Sciences 11: 2097–2108. 20559504 10.3390/ijms11052097 PMC2885096

[29] Lei FM, Qu YH, Lu JL, Liu Y, Yin ZH (2003) Conservation on diversity and distribution patterns of endemic birds in China. Biodiversity and Conservation 12: 239–254.

[30] Lei FM, Wei GA, Zhao HF, Yin ZH, Lu JL (2007) China subregional avian endemism and biodiversity conservation. Biodiversity and Conservation 16: 1119–1130.

[31] Sandel B, Arge L, Dalsgaard B, Davies RG, Gaston KJ, et al (2011) The influence of late quaternary climate-change velocity on species endemism. Science 334: 660–664. 21979937 10.1126/science.1210173

[32] Lei FM (2011) Global Endemism Needs Spatial Integration. Science 335: 284–285. 10.1126/science.335.6066.284-b 22267791

[33] Mittermeier RA, Gil PR, Mittermeier CG (1997) Megadiversity: Earth's Biologically. Wealthiest Nations. CEMEX/Agru paciaon, Sierra Madre, Mexico City, Mexico.

[34] Myers N, Mittermeier RA, Mittermeier CG, Da Fonseca GAB, Kent J (2000) Biodiversity hotspots for conservation priorities. Nature 403: 853–858. 10706275 10.1038/35002501

[35] Gómez A, Carvalho GR, Lunt DH (2000) Phylogeography and regional endemism of a passively dispersing zooplankter: mitochondrial DNA variation in rotifer resting egg banks. Proceedings of the Royal Society of London Series B: Biological Sciences 267: 2189–2197. 11413632 10.1098/rspb.2000.1268 PMC1690794

[36] Carnaval AC, Moritz C (2008) Historical climate modelling predicts patterns of current biodiversity in the Brazilian Atlantic forest. Journal of Biogeography 35: 1187–1201.

[37] Carnaval AC, Hickerson MJ, Haddad CFB, Rodrigues MT, Moritz C (2009) Stability predicts genetic diversity in the Brazilian Atlantic forest hotspot. Science 323: 785. 19197066 10.1126/science.1166955

[38] Excoffier L, Foll M, Petit RJ (2009) Genetic consequences of range expansions. Annual Review of Ecology, Evolution, and Systematics 40: 481–501.

[39] Dickinson EC (2003) The Howard and Moore complete checklist of the birds of the world. Christopher Helm.

[40] del Hoyo J, Elliot A, Christie D (2007) Handbook of the birds of the world. Vol. 12, picathartes to tits and chickadees. Lynx Edicions.

[41] Barraclough TG, Vogler AP (2000) Detecting the geographical pattern of speciation from species-level phylogenies. American Naturalist 155: 419–434. 10.1086/303332 10753072

[42] Sandel B, Arge L, Dalsgaard B, Davies RG, Gaston KJ, et al (2011) Response to global Endemism Needs Spatial Integration. Science 335: 285–286.

[43] Dai C, Zhao N, Wang W, Lin C, Gao B, et al (2011) Profound Climatic Effects on Two East Asian Black-Throated Tits (Ave: Aegithalidae), Revealed by Ecological Niche Models and Phylogeographic Analysis. PLoS One 6: e29329. 22195047 10.1371/journal.pone.0029329 PMC3241714

[44] Sorenson MD, Ast JC, Dimcheff DE, Yuri T, Mindell DP (1999) Primers for a PCR-based approach to mitochondrial genome sequencing in birds and other vertebrates. Molecular Phylogenetics and Evolution 12: 105–114. 10381314 10.1006/mpev.1998.0602

[45] Thompson JD, Gibson TJ, Plewniak F, Jeanmougin F, Higgins DG (1997) The CLUSTAL_X windows interface: flexible strategies for multiple sequence alignment aided by quality analysis tools. Nucleic Acids Research 25: 4876–4882. 9396791 10.1093/nar/25.24.4876 PMC147148

[46] Librado P, Rozas J (2009) DnaSP v5: a software for comprehensive analysis of DNA polymorphism data. Bioinformatics 25: 1451–1452. 19346325 10.1093/bioinformatics/btp187

[47] Posada D, Crandall KA (1998) Modeltest: testing the model of DNA substitution. Bioinformatics 14: 817–818. 9918953 10.1093/bioinformatics/14.9.817

[48] Posada D, Buckley TR (2004) Model selection and model averaging in phylogenetics: advantages of Akaike information criterion and Bayesian approaches over likelihood ratio tests. Systematic Biology 53: 793–808. 15545256 10.1080/10635150490522304

[49] Guindon S, Gascuel O (2003) A simple, fast, and accurate algorithm to estimate large phylogenies by N maximum likelihood. Systematic Biology 52: 696–704. 14530136 10.1080/10635150390235520

[50] Ronquist F, Huelsenbeck JP (2003) MrBayes 3: Bayesian phylogenetic inference under mixed models. Bioinformatics 19: 1572–1574. 12912839 10.1093/bioinformatics/btg180

[51] Excoffier LG, Laval G, Schneider S (2005) Arlequin ver. 3.0: An integrated software package for population genetics data analysis. Evolutionary Bioinformatics Online 1: 47–50. PMC2658868 19325852

[52] Tamura K, Dudley J, Nei M, Kumar S (2007) MEGA4: molecular evolutionary genetics analysis (MEGA) software version 4.0. Molecular Biology and Evolution 24: 1596–1599. 17488738 10.1093/molbev/msm092

[53] Tamura K, Nei M (1993) Estimation of the number of nucleotide substitutions in the control region of mitochondrial DNA in humans and chimpanzees. Molecular Biology and Evolution 10: 512–526. 8336541 10.1093/oxfordjournals.molbev.a040023

[54] Lovette IJ (2004) Mitochondrial dating and mixed-support for the “2% rule” in birds. The Auk 121: 1–6.

[55] Weir JT, Schluter D (2008) Calibrating the avian molecular clock. Molecular Ecology 17: 2321–2328. 18422932 10.1111/j.1365-294X.2008.03742.x

[56] Tajima F (1983) Evolutionary relationship of DNA sequences in finite populations. Genetics 105: 437–460. 6628982 10.1093/genetics/105.2.437 PMC1202167

[57] Fu YX (1997) Statistical tests of neutrality of mutations against population growth, hitchhiking and background selection. Genetics 147: 915–925. 9335623 10.1093/genetics/147.2.915 PMC1208208

[58] Drummond AJ, Rambaut A, Shapiro B, Pybus OG (2005) Bayesian coalescent inference of past population dynamics from molecular sequences. Molecular Biology and Evolution 22: 1185–1192. 15703244 10.1093/molbev/msi103

[59] Drummond AJ, Rambaut A (2007) BEAST: Bayesian evolutionary analysis by sampling trees. BMC Evolutionary Biology 7: 214. 17996036 10.1186/1471-2148-7-214 PMC2247476

[60] Drummond AJ, Ho SYW, Phillips MJ, Rambaut A (2006) Relaxed phylogenetics and dating with confidence. PLoS Biology 4: 699–710. 10.1371/journal.pbio.0040088 PMC1395354 16683862

[61] Rambaut A, Drummond AJ (2005) Tracer v1. 3. Available from http://beast.bio.ed.ac.uk/Tracer.

[62] Martínez-Meyer E, Peterson AT, Hargrove WW (2004) Ecological niches as stable distributional constraints on mammal species, with implications for Pleistocene extinctions and climate change projections for biodiversity. Global Ecology and Biogeography 13: 305–314.

[63] Martínez-Meyer E, Peterson AT (2006) Conservatism of ecological niche characteristics in North American plant species over the Pleistocene-to-Recent transition. Journal of Biogeography 33: 1779–1789.

[64] Peterson AT, Sober J, Sanchez-Cordero V (1999) Conservatism of ecological niches in evolutionary time. Science 285: 1265–1267. 10455053 10.1126/science.285.5431.1265

[65] Phillips SJ, Anderson RP, Schapire RE (2006) Maximum entropy modeling of species geographic distributions. Ecological Modelling 190: 231–259.

[66] Elith J, Graham CH, Anderson RP, Dudik M, Ferrier S, et al (2006) Novel methods improve prediction of species’ distributions from occurrence data. Ecography 29: 129–151.

[67] Hernandez PA, Graham CH, Master LL, Albert DL (2006) The effect of sample size and species characteristics on performance of different species distribution modeling methods. Ecography 29: 773–785.

[68] Hijmans RJ, Graham CH (2006) The ability of climate envelope models to predict the effect of climate change on species distributions. Global Change Biology 12: 2272–2281.

[69] Hijmans R, Cameron S, Parra J, Jones P, Jarvis A (2005) Very high resolution interpolated climate surfaces for global land areas. International Journal of Climatology 25: 1965–1978.

[70] Collins WD, Blackmon M, Bitz C, Bonan G, Bretherton CS, et al (2004) The community climate system model version 3 (CCSM3). Journal of Climate 19: 2122–2143.

[71] Hasumi H, Emori S (2004) K-1 coupled GCM (MIROC) description. Center for Climate System Research, University of Tokyo, Tokyo.

[72] Swets J (1988) Measuring the accuracy of diagnostic systems. Science 240: 1285. 3287615 10.1126/science.3287615

[73] Peterson AT, Papeş M, Eaton M (2007) Transferability and model evaluation in ecological niche modeling: A comparison of GARP and Maxent. Ecography 30: 550–560.

[74] Warren DL, Glor RE, Turelli M (2008) Environmental niche equivalency versus conservatism: Quantitative approaches to niche evolution. Evolution 62: 2868–2883. 18752605 10.1111/j.1558-5646.2008.00482.x

[75] Schoener TW (1968) Anolis lizards of Bimini: resource partitioning in a complex fauna. Ecology, 49, 704–726.

[76] Song G, Qu Y, Yin Z, Li S, Liu N, et al (2009) Phylogeography of the Alcippe morrisonia (Aves: Timaliidae): long population history beyond late Pleistocene glaciations. BMC Evolutionary Biology 9: 143–153. 19558699 10.1186/1471-2148-9-143 PMC2714695

[77] Li SH, Li JW, Han LX, Yao CT, Shi H, et al (2006) Species delimitation in the Hwamei Garrulax canorus. Ibis 148: 698–706.

[78] Gao LM, Moller M, Zhang XM, Hollingsworth ML, Liu J, et al (2007) High variation and strong phylogeographic pattern among cpDNA haplotypes in Taxus wallichiana (Taxaceae) in China and North Vietnam. Molecular Ecology 16: 4684–4698. 17908214 10.1111/j.1365-294X.2007.03537.x

[79] Qiu YX, Guan BC, Fu CX, Comes HP (2009) Did glacials and/or interglacials promote allopatric incipient speciation in east Asian temperate plants? Phylogeographic and coalescent analyses on refugial isolation and divergence in Dysosma versipellis. Molecular Phylogenetics and Evolution 51: 281–293. 19405195 10.1016/j.ympev.2009.01.016

[80] Cheng J, Liu X, Gao Z, Tang D, Yue J (2001) Effect of the Tibetan Plateau uplifting on the geological environment of the Yunnan Plateau. Geoscience 15: 290–296.

[81] Yang S, Dong H, Lei F (2009) Phylogeography of regional fauna on the Tibetan Plateau: A review. Progress in Natural Science 19: 789–799.

[82] Quade J, Cerling TE, Bowman JR (1989) Development of Asian monsoon revealed by marked ecological shift during the latest Miocene in northern Pakistan. Nature 342: 163.

[83] Wan SM, Li AC, Clift PD, Jiang HY (2006) Development of the East Asian summer monsoon: Evidence from the sediment record in the South China Sea since 8.5 Ma. Palaeogeography, Palaeoclimatology, Palaeoecology 241: 139–159.

[84] Li JJ, Fang XM (1999) Uplift of Qinghai-Tibetan Plateau and environmental change. Chinese Science Bulletin 44: 2217–2224.

[85] Webb IT, Bartlein PJ (1992) Global changes during the last 3 million years: climatic controls and biotic responses. Annual Review of Ecology and Systematics 23: 141–173.

[86] Dynesius M, Jansson R (2000) Evolutionary consequences of changes in species’ geographical distributions driven by Milankovitch climate oscillations. Proceedings of the National Academy of Sciences, USA 97: 9115–9120. 10.1073/pnas.97.16.9115 PMC16831 10922067

[87] Lovette IJ (2005) Glacial cycles and the tempo of avian speciation. Trends in Ecology and Evolution 20: 57–59. 16701342 10.1016/j.tree.2004.11.011

[88] Juan C, Emerson BC, Orom P, Hewitt GM (2000) Colonization and diversification: towards a phylogeographic synthesis for the Canary Islands. Trends in Ecology and Evolution 15: 104–109. 10675925 10.1016/s0169-5347(99)01776-0

[89] Li J, Yeung CKL, Tsai P, Lin RC, Yeh C, et al (2010) Rejecting strictly allopatric speciation on a continental island: prolonged postdivergence gene flow between Taiwan (Leucodioptron taewanus, Passeriformes Timaliidae) and Chinese (L. canorum canorum) hwameis. Molecular Ecology 19: 494–507. 20070521 10.1111/j.1365-294X.2009.04494.x

[90] Voris HK (2000) Special paper 2: Maps of pleistocene sea levels in Southeast Asia: Shorelines, river systems and time durations. Journal of Biogeography 27: 1153–1167.

[91] Yu G, Chen X, Ni J, Cheddadi R, Guiot J, et al (2000) Palaeovegetation of China: a pollen data-based synthesis for the mid-Holocene and last glacial maximum. Journal of Biogeography 27: 635–664.

[92] Harrison SP, Yu G, Takahara H, Prentice IC (2001) Palaeovegetation: diversity of temperate plants in East Asia. Nature 413: 129–130. 11557970 10.1038/35093166

[93] Gibbard PL, Boreham S, Cohen KM, Moscariello A (2007) Global chronostratigraphical correlation table for the last 2.7 million years. Subcommission on Quaternary Stratigraphy, Department of Geography, University of Cambridge, Cambridge, England.

[94] Zou F, Lim HC, Marks BD, Moyle RG, Sheldon FH (2007) Molecular phylogenetic analysis of the Grey-cheeked Fulvetta (Alcippe morrisonia) of China and Indochina: a case of remarkable genetic divergence in a ‘species’. Molecular Phylogenetics and Evolution 44: 165–174. 17300964 10.1016/j.ympev.2006.12.004

[95] Macarthur R H, Wilson E O (1967) The theory of island biogeography. Princeton University Press, Princeton, New Jersey, USA.

[96] Fjeldså J, Lambin E, Mertens B (1999) The relationship of species richness and endemism to ecoclimatic stability - a case study comparing distributions of Andean birds with remotely sensed environmental data. Ecography 22: 63–78.

[97] Klicka J, Zink RM (1997) The importance of recent ice ages in speciation: a failed paradigm. Science 277: 1666–1669.

[98] Johnson NK, Cicero C (2004) New mitochondrial DNA data affirm the importance of Pleistocene speciation in North American birds. Evolution 58: 1122–1130. 15212392 10.1111/j.0014-3820.2004.tb00445.x

[99] Weir JT, Schluter D (2007) The latitudinal gradient in recent speciation and extinction rates of birds and mammals. Science 315: 1574–1576. 17363673 10.1126/science.1135590

[100] Li SH, Yeung CK, Feinstein J, Han L, Le MH, et al (2009) Sailing through the Late Pleistocene: unusual historical demography of an East Asian endemic, the Chinese Hwamei(Leucodioptron canorum canorum), during the last glacial period. Molecular Ecology 18: 622–633. 19215583 10.1111/j.1365-294X.2008.04028.x

[101] Huang Z, Liu N, Liang W, Zhang Y, Liao X, et al (2010) Phylogeography of Chinese bamboo partridge, Bambusicola thoracica thoracica (Aves: Galliformes) in south China: Inference from mitochondrial DNA control-region sequences. Molecular Phylogenetics and Evolution 56: 273–280. 20132900 10.1016/j.ympev.2010.01.028

[102] Qian H, Ricklefs RE (2000) Large-scale processes and the Asian bias in species diversity of temperate plants. Nature 407: 180–182. 11001054 10.1038/35025052

[103] Qian H, Ricklefs RE (2001) Diversity of temperate plants in East Asia – reply. Nature 413: 130. 10.1038/35093166 11557970

[104] Qiu YX, Fu CX, Comes HP (2011) Plant molecular phylogeography in China and adjacent regions: Tracing the genetic imprints of Quaternary climate and environmental change in the world’s most diverse temperate flora. Molecular Phylogenetics and Evolution 59: 225–244. 21292014 10.1016/j.ympev.2011.01.012

[105] Wollenberg KC, Vieites DR, Van Der Meijden A, Glaw F, Cannatella DC, et al (2008) Patterns of endemism and species richness in malagasy cophyline frogs support a key role of mountainous areas for speciation. Evolution 62: 1890–1907. 18485110 10.1111/j.1558-5646.2008.00420.x

[106] Slatkin M (1987) Gene flow and the geographic structure of natural populations. Science 236: 787–792. 3576198 10.1126/science.3576198

[107] Barton NH (1979) Gene flow past a cline. Heredity 43: 333–339.

[108] Wiens JJ (2004) Speciation and ecology revisited: phylogenetic niche conservatism and the origin of species. Evolution 58: 193–197. 15058732 10.1111/j.0014-3820.2004.tb01586.x

[109] Liu H, Xing Q, Ji Z, Xu L, Tian Y (2003) An outline of Quaternary development of Fagus forest in China: palynological and ecological perspectives. Flora 198: 249–259.

