# Supplementary material for: Interpreting the Process behind Endemism in China by Integrating the Phylogeography and Ecological Niche Models of the Stachyridopsis ruficeps
Source: PLoS One. 2012 Oct 2;7(10):e46761. doi: 10.1371/journal.pone.0046761 (PMC3462788; doi:10.1371/journal.pone.0046761)
Supplement: Table S1 — The nested primers and thermocycling program for the three Nepal specimens. (DOC) [file pone.0046761.s001.doc]

**Table S1** The nested primers and thermocycling program for the three Museum specimens.

| Genes |  | Primers name | Sequence 5’-3’ |
| --- | --- | --- | --- |
| COI | 1 | L6615 | CCTCTATAAAAAGGTCTACAGCC |
|  |  | COIB114 | TGTAGATTTGATCGTCTCCTAG |
|  | 2 | COIF62 | TGCATGAGCCGGAATAGTTGGT |
|  |  | COIB279 | GGAAGGAGGGTGGGAGTAGTCA |
|  | 3 | COIF270 | TCCCACGAATAAACAACA |
|  |  | COIB549 | GCAGAGATAGGAGGAGAAGGAC |
|  | 4 | COIF536 | TCTGTTTGTGTGATCAGTAC |
|  |  | COIB870 | ATGTGAAGTATGCTCGGGTGTC |
|  | 5 | COIF811 | AGTCTGAGCAATACTCTCTATC |
|  |  | COIB1139 | AGTGGGTCAATCCTGCTAG |
|  | 6 | COIF1105 | TACTACGTAGTAGCCCACTTCCAC |
|  |  | H7956 | GGGTAGTCGGAGTATCGACG |
| Cyt b | 1 | CYTBL162 | CTCTCCTAGGCGTCTGC |
|  |  | CYTBH405 | CAAAGGCTGTGGCTATGAGTGTG |
|  | 2 | CYTBL373 | TACGGCTCATACCTAAACAAAGAA |
|  |  | CYTBH598 | GCCTGCGATGACGAATGG |
|  | 3 | CYTBL532 | TCGGACAAACCTTAGTAGAATGAG |
|  |  | CYTBH862 | AGAATGGCGTAGGCGAATAGGAA |
|  | 4 | CYTBL841 | CCCCTAGCAACACCACCAC |
|  |  | CYTBLOA | ATAGTTTGAGTATTTTGTTCTCTA |

Thermocycling program: an initial denaturation at 94°C for 5 min, followed by 40 cycles of 94°C for 40 s, 50°C for 35 s, and 72°C for 40 s, plus a final extension at 72°C for 8 min.
