# Supplementary material for: Interpreting the Process behind Endemism in China by Integrating the Phylogeography and Ecological Niche Models of the Stachyridopsis ruficeps
Source: PLoS One. 2012 Oct 2;7(10):e46761. doi: 10.1371/journal.pone.0046761 (PMC3462788; doi:10.1371/journal.pone.0046761)
Supplement: Table S2 — Results of the hierarchical analyses of genetic variance (AMOVA). (DOC) [file pone.0046761.s002.doc]

**Table S2** Results of hierarchical analyses of genetic variance (AMOVA).

Among Among pops Within

groups within groups pops Percentage of variation(%)

Groups

Among Among pops Within *F*_CT_ *F*_SC_ *F*_ST_ groups within groups pops

| [All] |  |  | 0.83*** |  | 83.73 | 16.27 |
| --- | --- | --- | --- | --- | --- | --- |
| [Southwest] [Xizang] [Taiwan] [Hainan] [Southeast] [Central] | 0.89*** | 0.14*** | 0.90*** | 88.89 | 1.51 | 9.60 |
| [bhamoensis] [davidi] [ruficeps] [praecognita] [goodsoni] | 0.81*** | 0.51*** | 0.91*** | 81.25 | 9.63 | 9.12 |
| [Taiwan] [Hainan] [Mainland] | 0.49** | 0.78*** | 0.89*** | 49.16 | 39.82 | 11.02 |

***P <* 0.01, ****P <* 0.0010
