# Supplementary material for: Interpreting the Process behind Endemism in China by Integrating the Phylogeography and Ecological Niche Models of the Stachyridopsis ruficeps
Source: PLoS One. 2012 Oct 2;7(10):e46761. doi: 10.1371/journal.pone.0046761 (PMC3462788; doi:10.1371/journal.pone.0046761)
Supplement: Table S3 — Corrected mean lineage distances and divergence times of the main lineages and the genetic variation within each lineage. (DOC) [file pone.0046761.s003.doc]

**Table S3** Corrected mean lineage distance estimated by Tajima-Nei model (below diagonal) and approximate divergence time of the main lineages (upper diagonal). Genetic variation within each lineage is shown in the diagonal.

| **Lineage** | **A1** | **A2** | **B** | **C** | **D** | **E** |
| --- | --- | --- | --- | --- | --- | --- |
| **A1** | 0.0005 | 0.373 | 2.243 | 2.51 | 2.51 | 2.61 |
| **A2** | 0.007 | 0.005 | 2.35 | 2.56 | 2.56 | 2.72 |
| **B** | 0.042 | 0.044 | 0.004 | 1.60 | 1.38 | 1.76 |
| **C** | 0.047 | 0.048 | 0.03 | 0.004 | 0.267 | 0.694 |
| **D** | 0.047 | 0.048 | 0.026 | 0.005 | 0.003 | 0.587 |
| **E** | 0.049 | 0.051 | 0.033 | 0.013 | 0.011 | 0.003 |
